# Clustering Based Physical-Layer Authentication in Edge Computing Systems with Asymmetric Resources

**DOI:** 10.3390/s19081926

**Published:** 2019-04-24

**Authors:** Yi Chen, Hong Wen, Jinsong Wu, Huanhuan Song, Aidong Xu, Yixin Jiang, Tengyue Zhang, Zhen Wang

**Affiliations:** 1 National Key Laboratory of Science and Technology on Communications, University of Electronic Science and Technology of China, Chengdu 611731, China; chenyi1309@126.com; 2 School of Aeronautics and Astronautics, University of Electronic Science and Technology of China, Chengdu 611731, China; huanhuansong@126.com (H.S.); uestczty@163.com (T.Z.); hswinston716@163.com (Z.W.); 3 Department of Electrical Engineering, Universidad de Chile, Santiago 833-0072, Chile; 4 EPRI, China Southern Power Grid Co., Ltd., Guangzhou 510080, China; xuad@csg.cn (A.X.); jiangyx@csg.cn (Y.J.)

**Keywords:** edge computing, clustering, physical-layer authentication, lightweight cipher, channel state information, lightweight authentication

## Abstract

In this paper, we propose a clustering based physical-layer authentication scheme (CPAS) to overcome the drawback of traditional cipher-based authentication schemes that suffer from heavy costs and are limited by energy-constrained intelligent devices. CPAS is a novel cross-layer secure authentication approach for edge computing system with asymmetric resources. The CPAS scheme combines clustering and lightweight symmetric cipher with physical-layer channel state information to provide two-way authentication between terminals and edge devices. By taking advantage of temporal and spatial uniqueness in physical layer channel responses, the non-cryptographic physical layer authentication techniques can achieve fast authentication. The lightweight symmetric cipher initiates user authentication at the start of a session to establish the trust connection. Based on theoretical analysis, the CPAS scheme is secure and simple, but there is no trusted party, while it can also resist small integer attacks, replay attacks, and spoofing attacks. Besides, experimental results show that the proposed scheme can boost the total success rate of access authentication and decrease the data frame loss rate, without notable increase in authentication latencies.

## 1. Introduction

**Figure 1:**
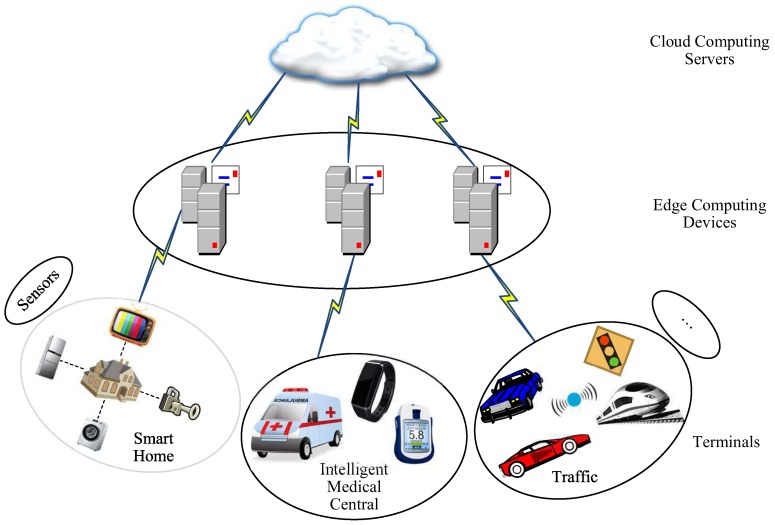
A simplified model of edge computing system.The edge computing system consists of edge computing devices who are usually specific high-end servers with powerful central processing unit, larger memory and storage, and various terminals that usually have limited resources [3] (such as limited computation power, battery, memory and bandwidth).

With the rapid development of Internet of things (IoT) technologies, various intelligent terminals (devices) have penetrated into our daily lives and works. As is well known, the traditional cloud computing system has some inherent limitations, namely real-time control incompetence [1], heavy network traffic, cloud data privacy insecurity, and so on. Luckily for us, the edge computing paradigm can also meet the key industrial requirements (such as instant links, real-time business, low latency and jitter, data security and privacy protection, and so on) by building small edge data centers [2]. As shown in Figure 1, the edge computing system consists of edge devices (edge servers) who are usually specific high-end servers with powerful central processing unit (CPU), larger memory and storage, and various terminals that usually have limited resources [3] (such as limited computation power, battery, memory, and bandwidth) due to cost constraints. Thus, it is vulnerable for IoT devices to be attacked by hackers or illegal users (such as replay, impersonation, eavesdropping, tampering, and so on) due to asymmetric resources. Identity authentication for communication participants (edge devices and terminals) is the basis and key to information security and privacy protection. Once the authentication system crashes, the whole system will be insecure. Traditional cryptographic ciphers can be divided into two categories, symmetric and asymmetric ciphers. Some of conventional symmetric ciphers are AES, DES or 3DES, and so on. RSA (Rivest, Shamir, and Adleman) and ECC (Elliptic Curve Cryptography) are the common asymmetric algorithms. They have one thing in common, namely large key size, which makes encryption or decryption slow and increases the complexity [4]. However, resource-constrained terminals often fail to satisfy the large memory requirements to store the large key size. Due to the limited resources about terminals, it is not suitable to use traditional complex encryption algorithms to implement access authentication. Therefore, it is necessary to design a lightweight identity authentication program for edge computing systems with asymmetric resources.

To provide security for resource-constrained devices, many lightweight symmetric ciphers have been proposed, such as MCRYPTON, HIGHT, PRESENT, MIBS, Piccolo, KLEIN, and so on [5]. They are secure and relatively fast but with low costs, and usually use the same key for both encryption and decryption of data [4]. Additionally, non-cryptographic authentication mechanisms based on physical-layer characteristics have been proposed for information security and privacy protection of devices in recent years [6,7,8,9], which have higher levels of security [10]. The authentication technique of physical layer based on channel state information (CSI) is one of the non-cryptographic authentication mechanisms [11], which can augment traditional network security [12]. It is carried out via comparing the similarity of CSI [13,14], which has the physical-layer channel characteristics of spatial-temporal uniqueness and can be extracted from the received data frames. In recent years, there have been many physical layer authentication methods based on machine learning (ML) [15,16,17,18,19,20]. However, the ML based physical layer authentication approach needs a large number of samples to train the network, which is unrealistic for real-time application. For the authentication technique of physical layer CSI, many research results have also been obtained [12,13,14,21,22,23,24,25]. However, the authentication rates of these methods need to be improved for their applications. The authentication rate mainly relies on the accuracy of CSI and the determination of test threshold. Finding suitable method to set the threshold according to environment is the most important to get high authentication rate, especially dynamically setting the threshold. Therefore, this paper present a clustering based physical-layer authentication scheme (CPAS). The proposed approach is a tradeoff between the traditional schemes [12,13,14,22] and machine learning based methods [15,16,17,18,19,20] for complexity and authentication rate. The advantage of the CPAS scheme is that the proposed method can adjust the decision threshold adaptively by updating the physical-layer channel authentication model and can be performed under limited data frames in the beginning, which can support the fast access.

Clustering is the unsupervised classification of data items into clusters [26]. Cluster analysis with little or no prior knowledge includes advanced techniques across various fields [27]. It plays a significant role in many disciplines [28]. Many researchers have proposed clustering algorithms [29,30]. However, there is little research on physical-layer security using clustering techniques. Considering the idea of clustering, in this research paper, we propose a clustering based physical-layer authentication scheme (CPAS), which is a novel cross-layer secure authentication approach for edge computing system with asymmetric resources. The CPAS scheme combines clustering technique and lightweight symmetric cipher with physical-layer channel state information to achieve two-way authentication between edge devices and terminals. The edge device does not drop data frames directly when physical-layer channel authentication fails, but to activate upper layer authentication to verify the legality of the data frames, which can resist losing legitimate data frames but lead to some processing delay. Moreover, multiple channel state information are used to establish a physical layer channel authentication model in the CPAS scheme, which magnify the differences between the multiple channel state information, but no effect on the performance of authentication. Experimental results show that our proposed scheme can effectively improve the success rate of physical-layer channel authentication, total success rate of access authentication and decrease the data frame loss rate without significantly increasing processing time. It is not only secure but also simple and flexible, especially independent of a third party. In addition, our scheme could resist spoofing attacks, replay attacks and small integer attacks. It can significantly reduce the access authentication complexity and achieve greater security for the edge computing system with asymmetric resources.

We summarize our main contributions as follows.

- We propose the first CPAS scheme, which combines clustering and lightweight symmetric cipher with physical-layer channel state information firstly and can be employed to authenticate mutually between terminals and edge devices. We also show the detailed implementing procedures of the proposed scheme.
- We analyze the security of the proposed scheme and prove that it can resist small integer attacks, replay attacks, and spoofing attacks.
- The CPAS scheme is implemented in a real world environment based on MIMO-OFDM systems. We also show the impacts of adjusting parameters of clusters on the success rate of physical-layer channel authentication, the data frame loss rate, the total success rate of access authentication, and the time cost through experimental results demonstration.

The rest of this paper is organized as follows. Section 2 introduces the basic principles of physical layer channel authentication. The system model and proposed CPAS scheme are presented in Section 3. The security of the proposed scheme is analyzed in Section 4. In Section 5, the experiment results indicate that the proposed CPAS scheme is effective for authentication. We conclude this paper in Section 6.

## 2. Basic Principles of Physical Layer Channel Authentication

In this section, we briefly present the basic principles of physical-layer channel authentication and show the shortcomings of some authentication schemes.

Xiao et al. designed a physical-layer authentication scheme via exploiting the spatial variability of the radio channel response [13]. However, the proposed scheme in [13] has the disadvantage of authenticating the initial data frame that is usually assumed to be valid. In their scheme, the receivers need to estimate the radio channel response, shown below

(1) $$\begin{array}{c} {\underline{H}}_{k}={\left[H_{k}\left(f_{1}\right),\cdots ,\;H_{k}\left(f_{i}\right),\cdots ,\;H_{k}\left(f_{M}\right)\right]}^{T}, \end{array}$$

where *k* denotes the data frame index, $f_{i}=f_{0}+\left(\frac{i}{M}-\frac{1}{2}\right)W$, i=1,2,⋯,M, $f_{0}$ is the center measurement frequency, *W* is the measurement bandwidth, and *M* is the number of measurement frequency over the measurement bandwidth.

The receiver utilizes channel state information in two consecutive data frames, $H_{k-1}$ and $H_{k}$, and hypothesis testing to determine whether they come from the same sender or not. Hypothesis testing is the task of deciding which of the two hypotheses, $H_{0}$ or $H_{1}$, is true, when one is given the value of a random variable [22]. $H_{k-1}$ and $H_{k}$ can be estimated by ILS channel estimation method [23,24,25]. In the null hypothesis, $H_{0}$, the claimant user is the initial sender. The base station accepts this hypothesis if the test statistic *T* is below some threshold Γ. Otherwise, in the alternative hypothesis, $H_{1}$, the claimant is someone else. The notation “∼” is used to indicate accurate values without measurement errors, and thus have

(2) $$\begin{array}{c} \begin{array}{c} H_{0}:{\underline{\tilde{H}}}_{k}={\underline{\tilde{H}}}_{k-1}\\ H_{1}:{\underline{\tilde{H}}}_{k}\neq {\tilde{\underline{H}}}_{k-1} \end{array}. \end{array}$$

The inherent physical parameters of the multi-path fading channels were exploited to support continuous mutual authentication between wireless terminals by He et al. [22]. He et al. [22] used the information of both amplitude and phase in the channel signature to enhance the communication security. They employed three statistical channel signature information to strengthen physical security. However, in reality, the noisy power is unknown. Thus, the test statistic of channel responses is normalized as follows

(3) $$\begin{array}{c} \Lambda_{i}=\frac{K_{co}{\left\|\left({\underline{H}}_{k-i+1}\left(i\right)-{\underline{H}}_{k-i}\left(i\right)e^{j\varphi }\right)\right\|}^{2}}{{\left\|{\underline{H}}_{k-i}\left(i\right)\right\|}^{2}}, \end{array}$$

where “*i*” is an index, i=1,2,⋯,S, “*S*” is a positive integer, and S⩾1. Then, the cumulative summation of the log-likelihood ratio Λ is calculated as

(4) $$\begin{array}{c} \Lambda =K_{co\_S}\sum_{i=1}^{S}\Lambda_{i}\begin{array}{c} >H_{1}\\ <H_{0} \end{array}\Gamma , \end{array}$$

where $K_{co\_S}$ denotes the normalization factor to let the threshold value Γ∈[0,1]. When S>1, it is sequential probability ratio test (SPRT). A SPRT could compare ${\underline{\tilde{H}}}_{k}$ with all past records $\left({\underline{\tilde{H}}}_{i}\right)$, where i<k in some way. When S=1, it is a likelihood ratio test (LRT). The LRT only compares the estimation in the *k*th data frame $({\underline{\tilde{H}}}_{k})$ with that in the (k−1)th data frame $({\underline{\tilde{H}}}_{k-1})$.

## 3. System Model and Proposed Scheme

**Figure 2:**
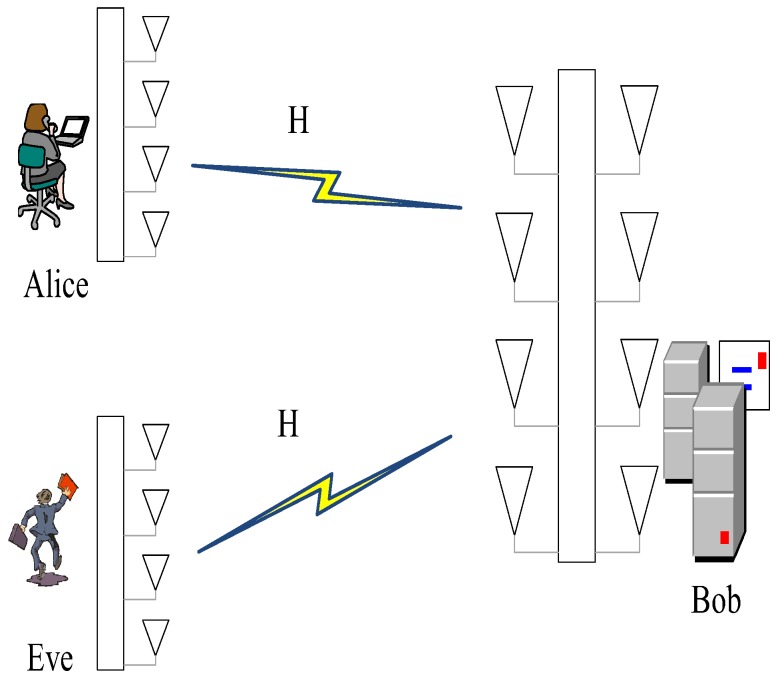
Scenario with Alice ($T_{E}$), Bob (ED), and Eve.

We consider the edge computing scenario shown in Figure 2, which consists of various terminals ($T_{E}$), also called Alice, and edge computing devices (ED), also called Bob. They want to exchange messages across a wireless link. It must be assured that the received data frames are all coming from the correct communication pair. Compared with the terminals with limited resources, edge devices are usually specific high-end servers with powerful CPUs, larger memory and storage units. Alice and Bob can perform authentication with each other via exchanging messages in the edge computing system with asymmetric resources. Their evil adversary, Eve, will play the part of an active opponent that injects undesirable messages into the medium in the expectations of spoofing Bob.

The proposed authentication scheme is divided into secret key sharing, initial authentication, physical-layer channel modeling, physical-layer channel authentication, lightweight cryptographic authentication, and model update of physical-layer channel authentication.

### 3.1. Secret Key Sharing

A secret key named Key is shared between Alice and Bob over a secure channel. This is not the essence of this article, thus we omit it here.

### 3.2. Initial Authentication

**Figure 3:**
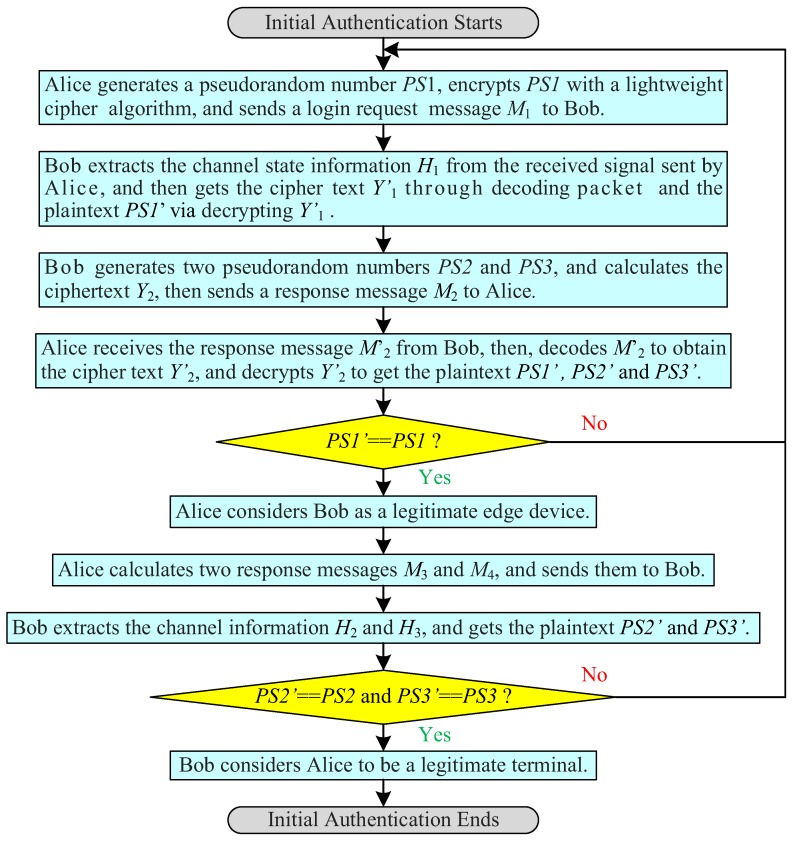
Process flowchart of initial authentication.

The initial authentication between the terminal and the edge computing device is completed through a lightweight cryptographic algorithm by using the same secret key. As shown in Figure 3, the initial full authentication phases are as follows:

(1) Alice generates a pseudorandom number PS1, and encrypts PS1 with a lightweight cryptographic algorithm to obtain ciphertext $Y_{1}=E_{\left(key\right)}\left(PS1\right)$, where $E_{\left(key\right)}\left(PS1\right)$ means that encrypting message, such as the random number PS1 in the parentheses by using a lightweight cryptographic algorithm and a secret key. Then, the terminal generates a login request message $M_{1}$ and sends it to the edge computing device, where the request message $M_{1}$ includes the ciphertext $Y_{1}$.
(2) Bob extracts the channel state information $H_{1}$ from the received signal sent by Alice, and then gets the ciphertext $Y_{1}^{\prime }$ from decoding data and the plaintext ${PS1}^{\prime }$ via decrypting $Y_{1}^{\prime }$ with the same lightweight cryptographic algorithm and secret key, where ${PS1}^{\prime }=D_{\left(key\right)}\left(Y_{1}^{\prime }\right)$, $D_{\left(key\right)}\left(Y_{1}^{\prime }\right)$ means that decrypting message, such as $Y_{1}^{\prime }$ in the parentheses via using a lightweight cryptographic algorithm and a secret key, and the channel information $H_{1}$ is a complex matrix of *m* rows and *n* columns.
(3) Bob generates two pseudorandom numbers PS2 and PS3, and calculates the ciphertext $Y_{2}=E_{\left(key\right)}({PS1}^{\prime }\|PS2\|PS3)$. Then, Bob sends a response message $M_{2}$ to Alice, where $M_{2}$ contains the ciphertext $Y_{2}$.
(4) Alice verifies the legitimacy of Bob. When Alice receives the response message $M_{2}^{\prime }$, it decodes $M_{2}^{\prime }$ to obtain the ciphertext $Y_{2}^{\prime }$, and then decrypts $Y_{2}^{\prime }$ to obtain the plaintext (${PS1}^{\prime }\|{PS2}^{\prime }\|{PS3}^{\prime }$) = $D_{\left(key\right)}\left(Y_{2}^{\prime }\right)$. If the ${PS1}^{\prime }$ is not equal to PS1, Bob is an illegal edge device and Alice cancels the login; otherwise, Alice considers Bob to be a legitimate edge computing device, calculates two response messages $M_{3}$ and $M_{4}$, and continuously sends them to the edge computing device, where $M_{3}$ includes ciphertext $Y_{3}=E_{\left(key\right)}\left({PS2}^{\prime }\right)$, and $M_{4}$ contains ciphertext $Y_{4}=E_{\left(key\right)}\left(PS3^{\prime }\right)$.
(5) Bob verifies the legitimacy of Alice. Bob extracts the channel information $H_{2}$ and $H_{3}$ from the received response messages $M_{3}$ and $M_{4}$ sent from Alice, and then gets the ciphertext $Y_{3}^{\prime }$ and $Y_{4}^{\prime }$ from decoding and the plaintext ${PS2}^{\prime }$ and ${PS3}^{\prime }$ by decrypting $Y_{3}^{\prime }$ and $Y_{4}^{\prime }$ with the same lightweight cryptographic algorithm and secret key, where ${PS2}^{\prime }=D_{\left(key\right)}\left(Y_{3}^{\prime }\right)$, ${PS3}^{\prime }=D_{\left(key\right)}\left(Y_{4}^{\prime }\right)$, the channel information $H_{2}$ extracted by Bob from $M_{3}$ and $H_{3}$ from $M_{4}$, $H_{2}$ and $H_{3}$ are complex matrices of *m* rows and *n* columns. If ${PS2}^{\prime }$ is equal to PS2 and ${PS3}^{\prime }$ is matching to PS3, Bob considers Alice as a legitimate terminal, and the initial authentication ends; otherwise, Alice is an illegal terminal and Bob cancels the login.

### 3.3. Physical-Layer Channel Modeling

**Figure 4:**
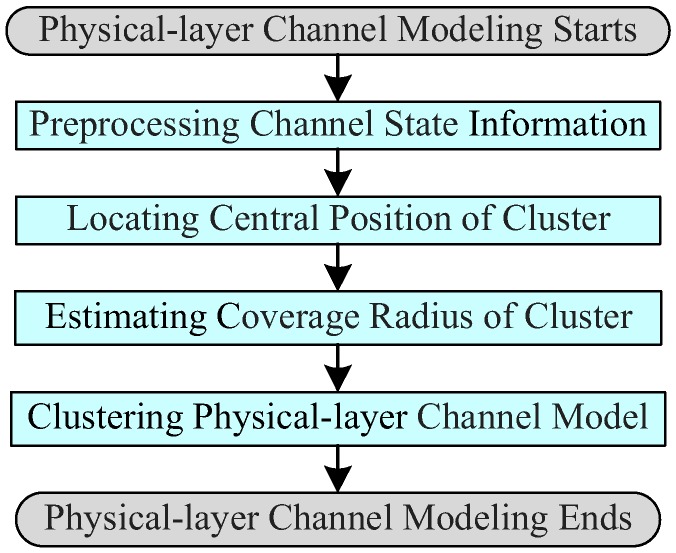
Process flowchart of physical-layer channel modeling.

**Figure 5:**
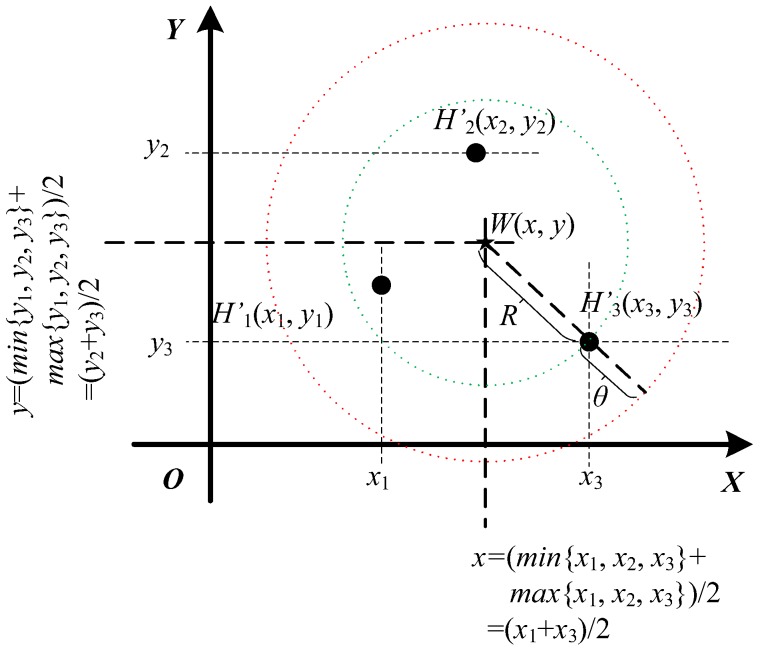
Principle map of physical-layer channel modeling.

Bob uses the channel state information, detected and estimated within the correlated time, for the physical-layer channel modeling. We consider the idea of clustering that is the task of organizing a set of objects into groups whose members are more similar to each other than to those in other groups (clusters). Bob needs at least three data frames to model the physical layer channel (organize a cluster of similar data frames). As shown in Figure 4, the physical-layer channel model consists of four parts: preprocessing channel state information, locating central position of cluster (channel model), estimating coverage radius of cluster, and clustering physical-layer channel model. Figure 5 is the detailed modeling principle of physical-layer channel.

(1) Preprocessing channel state information
  The channel information $H_{1}$, $H_{2}$, and $H_{3}$, which are extracted during the initial full authentication phase, are complex matrices of *m* rows and *n* columns, where *m* denotes the number of carriers, and *n* indicates the number of antennas. To obtain the statistical characteristics of channel information, we accumulate the absolute value of the real part and the imaginary part about the complex matrices, respectively. The statistical coordinates of channel information are named as $H_{1}^{\prime }\left(x_{1},y_{1}\right)$, $H_{2}^{\prime }\left(x_{2},y_{2}\right)$, and $H_{3}^{\prime }\left(x_{3},y_{3}\right)$, which are coordinate pairs on the complex plane.
(2) Locating central position of cluster
  After completing the previous sub-step, preprocessing channel information, the central position of cluster (channel model), named as W(x,y), is estimated by
  (5) $$\begin{array}{c} \left\{\begin{array}{c} x=\frac{min\left\{\begin{array}{c} x_{1},x_{2},x_{3} \end{array}\right\}+max\left\{\begin{array}{c} x_{1},x_{2},x_{3} \end{array}\right\}}{2}\\ y=\frac{min\left\{\begin{array}{c} y_{1},y_{2},y_{3} \end{array}\right\}+max\left\{\begin{array}{c} y_{1},y_{2},y_{3} \end{array}\right\}}{2} \end{array}\right., \end{array}$$
  where min{·} represents minimum value, while max{·} implies maximum value.
(3) Estimating coverage radius of cluster
  The Euclidean distances between the central position W(x,y) and the statistical position of channel information $H_{1}^{\prime }\left(x_{1},y_{1}\right)$, $H_{2}^{\prime }\left(x_{2},y_{2}\right)$, and $H_{3}^{\prime }\left(x_{3},y_{3}\right)$ are given by
  (6) $$\begin{array}{c} \left|\left|WH_{n}^{\prime }\right|\right|=\sqrt{{\left(x_{W}-x_{H_{n}^{\prime }}\right)}^{2}+{\left(y_{W}-y_{H_{n}^{\prime }}\right)}^{2}}, \end{array}$$
  where $\left\|WH_{n}^{\prime }\|(n=1,2,3\right)$ denotes the Euclidean distances between W(x,y) and $H_{1}^{\prime }$, $H_{2}^{\prime }$, and $H_{3}^{\prime }$, respectively. Then, the maximum Euclidean distance is taken as the radius (R) of cluster.
  (7) $$\begin{array}{c} R=max\left\{\begin{array}{c} \left|\left|WH_{1}^{\prime }\right|\right|,\left|\left|WH_{2}^{\prime }\right|\right|,\left|\left|WH_{3}^{\prime }\right|\right| \end{array}\right\}, \end{array}$$
  where *R* denotes the radius of cluster. Further, the coverage radius of channel model is obtained by
  (8) $$\begin{array}{c} dist=R+\theta , \end{array}$$
  where θ is the adjusting parameter of the coverage radius of channel model.
(4) Clustering physical-layer channel model
  When the central position and the coverage radius of channel model are determined, the categories of physical-layer channel model are defined as
  (9) $$\begin{array}{c} C_{i}=\{W_{i},dist_{i}\}, \end{array}$$
  where *i* indicates the index of terminal, and different $C_{i}$ is specified for a different cluster, i.e., a different terminal.

The physical-layer channel modeling is completed.

### 3.4. Physical-Layer Channel Authentication

**Figure 6:**
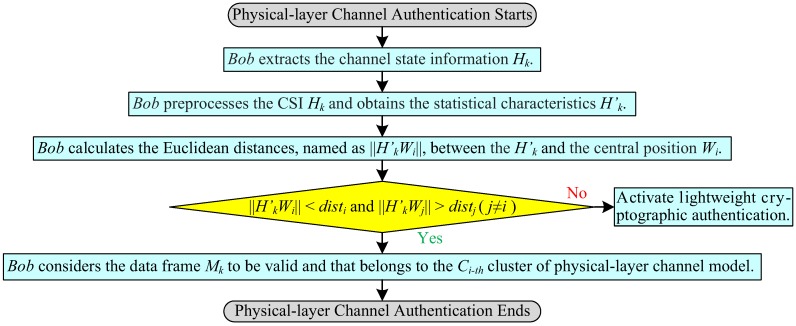
Process flowchart of physical-layer channel authentication.

**Figure 7:**
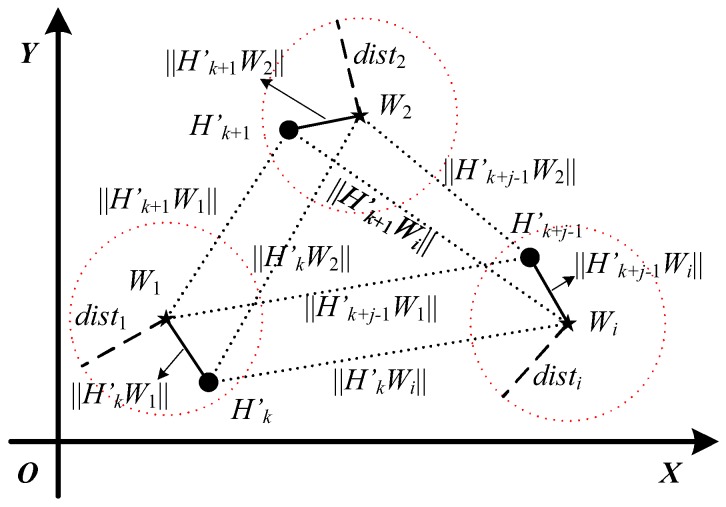
Principle map of physical-layer channel authentication.

When Bob receives a new data frame, it can directly verify the legality of the data frame according to the established physical-layer channel model. Figure 6 is the process flowchart of physical-layer channel authentication. The detailed authentication principle map of physical-layer channel is exhibited in Figure 7.

(1) Bob extracts the channel information $H_{k}$ from the received data frame $M_{k}$ sent from Alice, where, the channel information $H_{k}$ is a complex matrix of *m* rows and *n* columns, the data frame $M_{k}$ contains the cipher text $Y_{k}^{\prime }=E_{\left(key\right)}\left(PS2_{i}^{\prime }⊕PS3_{i}^{\prime }\right)$, “⨁” means XOR function, and the *k* indicates the index of data frame.
(2) Bob preprocesses the channel information $H_{k}$. To obtain the statistical characteristics $H_{k}^{\prime }\left(x_{k},y_{k}\right)$ of channel information, Bob accumulates the absolute value of the real part and the imaginary part of $H_{k}$, respectively. The statistical characteristics $H_{k}^{\prime }\left(x_{k},y_{k}\right)$ denote the coordinate pairs on the complex plane.
(3) Bob checks the validity of the data frame $M_{k}$. Firstly, Bob calculates the Euclidean distances, named as $\left\|H_{k}^{\prime }W_{i}\right\|$, between the $H_{k}^{\prime }$ and the central position $W_{i}$ of the cluster, respectively. Then, Bob compares the sizes of $\left\|H_{k}^{\prime }W_{i}\right\|$ and $dist_{i}$: when $\|H_{k}^{\prime }W_{i}\|<dist_{i}$(i∈S={1,2,⋯}) and $\|H_{k}^{\prime }W_{j}\|>dist_{j}$(∀j∈{j|j∈S,j≠i}), Bob considers the data frame $M_{k}$ to be valid and that belongs to the $C_{i\text{-}\mathrm{th}}$ cluster (physical-layer channel model); otherwise, Bob activates lightweight cryptographic authentication.

### 3.5. Lightweight Cryptographic Authentication

**Figure 8:**
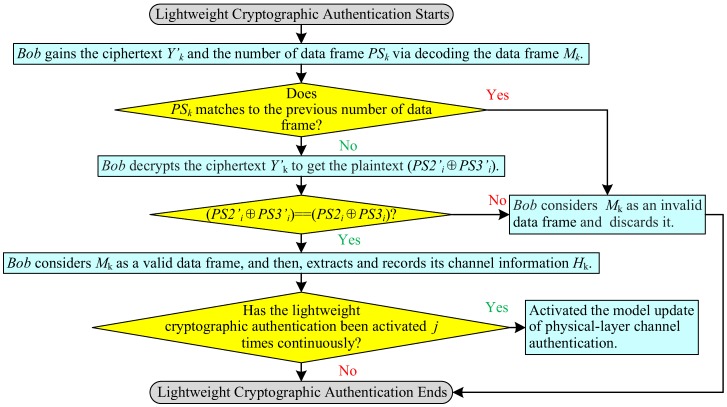
Process flowchart of lightweight cryptographic authentication.

During the non-initial authentication phase, if Bob cannot check the validity of the data frame $M_{k}$ coming from terminal through the physical-layer channel authentication, the lightweight cryptographic authentication will be activated. The process flowchart of lightweight cryptographic authentication is shown in Figure 8.

(1) Bob gains the ciphertext, $Y_{k}^{\prime }$, and the number of data frame, $PS_{k}$, which is also a pseudorandom number, via decoding the data frame $M_{k}$ sent from Alice, where $Y_{k}^{\prime }=E_{\left(key\right)}\left(PS2_{i}^{\prime }⊕PS3_{i}^{\prime }\right)$, and the length of the random number is determined according to the actual application scenario. If $PS_{k}$ matches the previous number of data frame, ED considers $M_{k}$ as a replayed packet and throws it away; otherwise, Bob goes to next step.
(2) Bob decrypts the ciphertext $Y_{k}^{\prime }$ to get the plaintext $\left(PS2_{i}^{\prime }⊕PS3_{i}^{\prime }\right)=D_{\left(key\right)}\left(Y_{k}^{\prime }\right)$.
(3) Bob checks the validity of the data frame $M_{k}$. If $\left(PS2_{i}^{\prime }⊕PS3_{i}^{\prime }\right)$ does not match $\left(PS2_{i}⊕PS3_{i}\right)$, the data frame $M_{k}$ is illegal and Bob discards it. If $\left(PS2_{i}^{\prime }⊕PS3_{i}^{\prime }\right)$ is equal to $\left(PS2_{i}⊕PS3_{i}\right)$, Bob considers $M_{k}$ as a valid data frame, and then extracts and records its channel information $H_{k}$. When Bob receives *j* data frames $\left\{M_{k}^{\prime },M_{k+1}^{\prime },\cdots ,M_{k+(j-1)}^{\prime }\right\}$, namely lightweight cryptographic authentication being activated *j* times continuously, the model update of physical-layer channel authentication will be activated.

### 3.6. Model Update of Physical-Layer Channel Authentication

**Figure 9:**
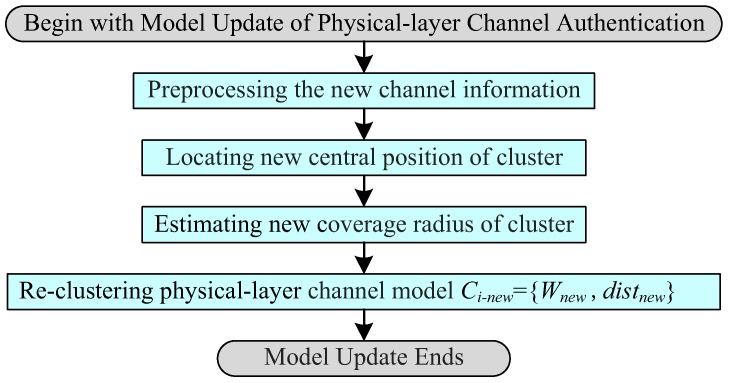
Process flowchart of model update of physical-layer channel authentication.

**Figure 10:**
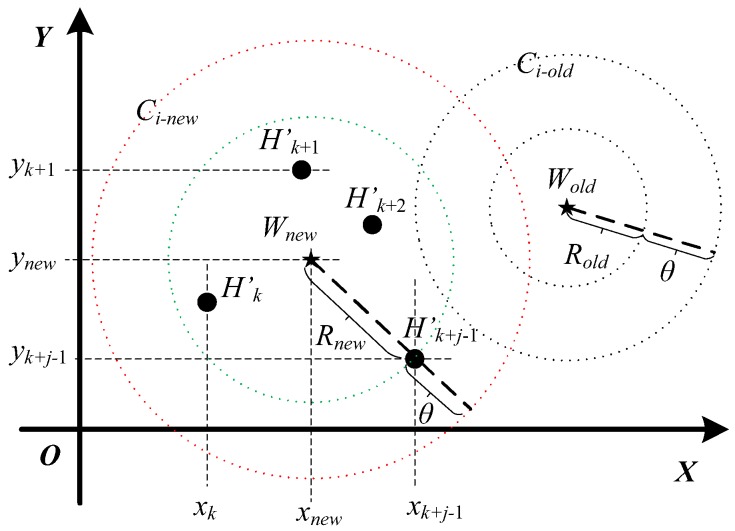
Principle map of model update of physical-layer channel authentication.

When lightweight cryptographic authentication is activated continuously *j* times to verify the validity of data frames $\left\{M_{k}^{\prime },M_{k+1}^{\prime },\cdots ,M_{k+(j-1)}^{\prime }\right\}$, Bob needs to update the physical-layer channel model for a renewed physical-layer authentication, where j⩾3. Figure 9 presents the process flowchart of model update of physical-layer channel authentication, which similar to the physical-layer channel modeling also contains four parts: preprocessing the new channel information, locating the new central position of the cluster, estimating the new coverage radius of the cluster, and re-clustering the physical-layer channel model. The detailed model update principle map of the physical-layer channel is displayed in Figure 10.

(1) Preprocessing the new channel information
  The sequences of channel information $H_{k}$, $H_{k+1}$, ⋯, $H_{k+(j-1)}$, which are extracted during the lightweight cryptographic authentication phase, are complex matrices of *m* rows and *n* columns. To obtain the statistical characteristics of channel information, we accumulate the absolute values of the real part and the imaginary part about the complex matrices, respectively. The statistical sequences of channel information are named as $H_{k}^{\prime }\left(x_{k},y_{k}\right)$, $H_{k+1}^{\prime }\left(x_{k+1},y_{k+1}\right)$, ⋯, $H_{k+j-1}^{\prime }\left(x_{k+j-1},y_{k+j-1}\right)$, which are coordinate pairs on the complex plane.
(2) Locating new central position of cluster
  After completing the previous sub-step, preprocessing the new channel information, the new central positions of physical-layer channel model, named as $W_{new}\left(x_{new},y_{new}\right)$, are estimated by Equation (10).
  (10) $$\begin{array}{c} \left\{\begin{array}{c} x_{new}=\frac{min\left\{x_{k},x_{k+1},\cdots ,x_{k+j-1}\right\}+max\left\{x_{k},x_{k+1},\cdots ,x_{k+j-1}\right\}}{2}\\ y_{new}=\frac{min\left\{y_{k},y_{k+1},\cdots ,y_{k+j-1}\right\}+max\left\{y_{k},y_{k+1},\cdots ,y_{k+j-1}\right\}}{2} \end{array}\right.. \end{array}$$
(3) Estimating new coverage radius of cluster
  The Euclidean distances between the new central position $W_{new}\left(x_{new},y_{new}\right)$ and the statistical sequences of channel information $H_{k}^{\prime }\left(x_{k},y_{k}\right)$, $H_{k+1}^{\prime }\left(x_{k+1},y_{k+1}\right)$, ⋯, $H_{k+j-1}^{\prime }\left(x_{k+j-1},y_{k+j-1}\right)$, are given by
  (11) $$\begin{array}{c} \left|\left|W_{new}H_{n}^{\prime }\right|\right|=\sqrt{{\left(x_{W_{new}}-x_{H_{n}^{\prime }}\right)}^{2}+{\left(y_{W_{new}}-y_{H_{n}^{\prime }}\right)}^{2}}, \end{array}$$
  where $\left\|W_{new}H_{n}^{\prime }\right\|$ (n=k, k+1, ⋯, k+j−1) denote the Euclidean distances. Then, the maximum Euclidean distance is taken as the new radius ($R_{new}$) of cluster.
  (12) $$\begin{array}{c} \begin{array}{c} R_{new}=max\{\|W_{new}H_{k}^{\prime }\|,\|W_{new}H_{k+1}^{\prime }\|,\cdots ,\|W_{new}H_{k+j-1}^{\prime }\|\} \end{array}, \end{array}$$
  where $R_{new}$ denotes the new radius of channel model. Further, the new coverage radius of cluster is obtained by
  (13) $$\begin{array}{c} dist_{new}=R_{new}+\theta , \end{array}$$
  where θ indicates the adjusting parameter of the coverage radius of channel model.
(4) Re-clustering physical-layer channel model
  When obtaining the new central position and the new coverage radius of channel model, the new cluster of physical-layer channel model is redefined as
  (14) $$\begin{array}{c} C_{i\text{-}new}=\{W_{i\text{-}new},dist_{i\text{-}new}\}. \end{array}$$

The model update of physical-layer channel authentication is completed.

## 4. Security Analysis

In this section, the proposed CPAS scheme is analyzed with respect to the security.

The proposed CPAS scheme can be used to authenticate mutually between terminals (Alice) and edge devices (Bob) for the edge computing system with asymmetric resources, despite the presence of Eve. In the CPAS scheme, the following security measures are adopted.

Firstly, the lightweight cipher algorithm is one of the security measures. A different lightweight cipher has a different security intensity. CPAS scheme can choose different lightweight cipher flexibly to encrypt data. Bob is usually a specific high-end server. He has the ability to withstand complex computations for different cryptographic algorithms. However, the appropriateness of Alice’s ciphers depend on her resources. Besides, there is no trusted party involved in the authentication process. Thus, the strategy is feasible for resource-constrained terminals, if lightweight cipher just keep them safe in a certain time, according to the requirement of application.

The second security measure is the use of pseudorandom number. The replay attacks and small integer attacks cannot be successful since the authentication messages are not the same every time. This is due to the use of dynamic authentication messages combined with a different pseudorandom number in every communication session and every data frame. In other words, the authentication packets generated in different valid phases are different, and the current authentication messages are valid only for the current authentication phase, since the pseudorandom number cannot be enumerated and the valid authentication messages cannot be generated in a period of data transmission. Thus far, researchers have proposed a lot of pseudorandom number generators [31,32,33,34]. The periods of different pseudorandom generators are different. For example, the Mersenne Twister MT19937 is a pseudorandom number generator and it has a large period of $2^{19937-1}$ [34]. Bob could still bear its computational complexity. In practical applications, users can choose the appropriate pseudorandom number generator according to their own needs. Thus, the exhaustive attacks and guessing attacks are also impossible, since the authentication messages are not the same every time.

In addition, physical-layer channel state information recognition technique is another security measure. It depends on the spatiotemporal uniqueness of physical-layer channel characteristics, which can be estimated from the received data frames. This can assist CPAS scheme to resist the spoofing attacks. Eve could not convince Bob that she is Alice.

Therefore, the proposed CPAS scheme not only can implement bidirectional authentication between Alice and Bob, but also can withstand replay attacks, small integer attacks, and spoofing attacks.

## 5. Performance

To examine the performances of the proposed CPAS scheme, we firstly simulated it in MATLAB under different signal-to-noise ratios (SNRs). In the simulations, we set the maximum Doppler shift of 15 Hz, the bandwidth of 1 MHz, the digital modulation method of QPSK, the number of subcarrier 128, the number of multi-paths 5, and 1000 times test.

**Figure 11:**
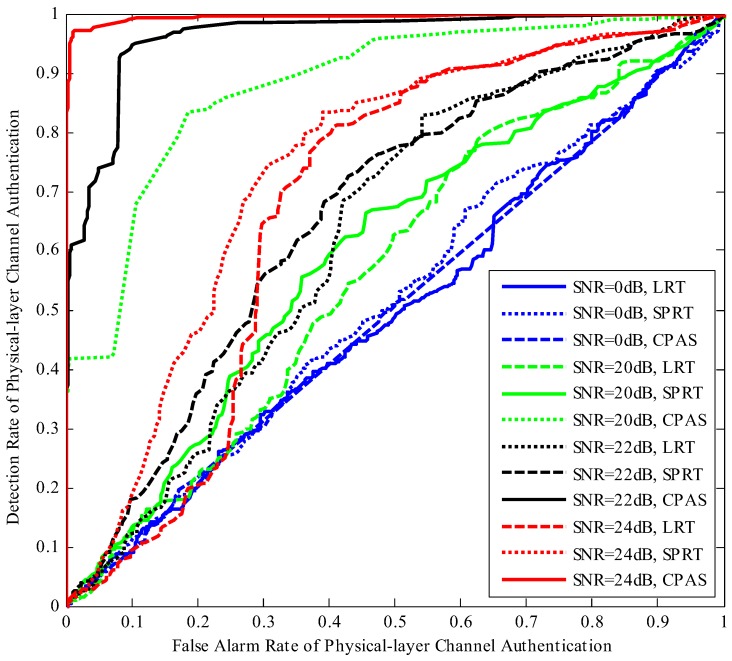
Comparisons of detection rate and false alarm rate under different SNRs.

Detection rate and false alarm rate of physical-layer channel authentication are two critical measurements. Detection rate of physical-layer channel authentication indicates the probability of illegal data frames detection and false alarm rate of physical-layer channel authentication denotes the probability of legitimate data frames detected as illegitimate. When the false alarm rate is smaller and detection rate is bigger, the authentication performance is better, where the false alarm rate of 0 and the detection rate of 1 are the ideal performances. Figure 11 depicts the diagram of detection rate and false alarm rate of physical layer channel authentication for different adjusting parameter θ. The proposed scheme was compared with the LRT and SPRT schemes. The performances of these schemes upgraded gradually with the increase of SNR, while the performance of CPAS was better than those of the other schemes under the same SNR.

The simulations in MATLAB demonstrated the advantages of the CPAS scheme, which was also implemented over universal software radio peripheral (USRP) platform [35,36,37]. Experiments were performed in an office room, which is 8 m long, 7.5 m wide, and 3 m high. Edge computing device was equipped with an 8×8 MIMO system. Terminal was equipped with a 2×2 MIMO system. They worked on the center frequency 3.5 GHz with the sub-bandwidth 2 MHz, the number of subcarrier 128, and the interval of sub-carriers 15.625 kHz. The wavelength of the transmission signal was about 0.086 m. The maximal transmitting power was 15 dBm and transmission gain 20 dB. The communication scheme was based on MIMO-OFDM (Multiple Input and Multiple Output—Orthogonal Frequency Division Multiplexing) and ILS (Improved-scaled Least Squares) was adopted to estimate channels. In our experiments, we employed RC4 algorithm to act a lightweight cryptographic algorithm, which is not the focus of this paper.

**Figure 12:**
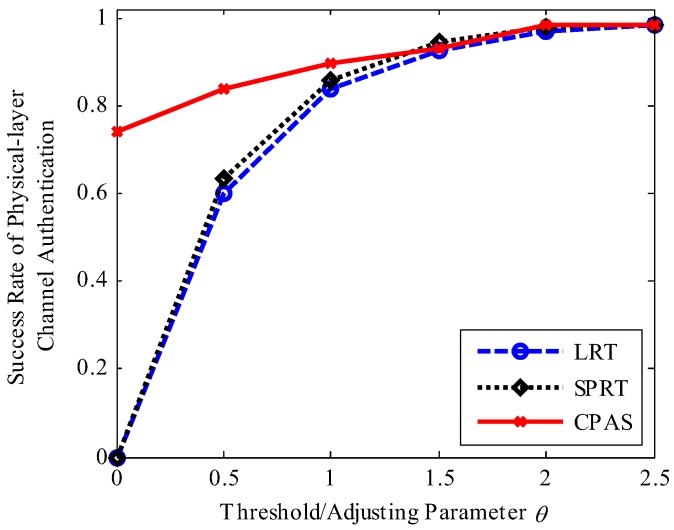
Success rate of physical-layer channel authentication versus threshold values or adjusting parameter θ. It shows the success rate of physical-layer channel authentication of different schemes at different θ.

**Figure 13:**
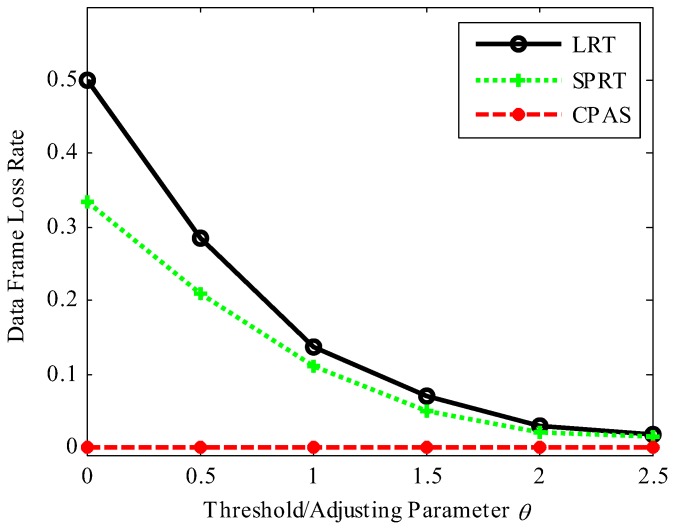
Data frame loss rate.

**Figure 14:**
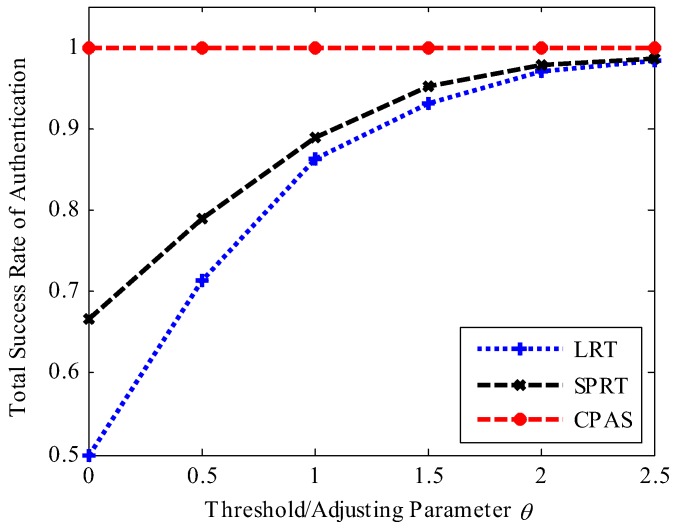
Total authentication success rate of different authentication scheme.

**Figure 15:**
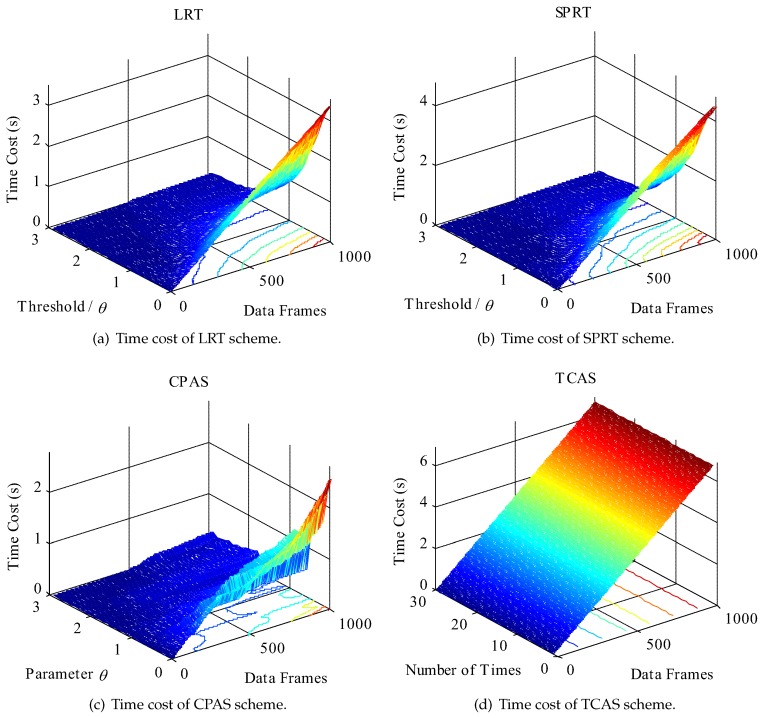
Three dimensional plots of time cost. Note that RC4 algorithm was employed to act a lightweight cryptographic algorithm in the experiments: (**a**) the time cost of LRT scheme; (**b**) the time cost of SPRT scheme; (**c**) the time cost of the proposed scheme, CPAS; and (**d**) the time cost of the traditional cipher authentication scheme, TCAS.

**Figure 16:**
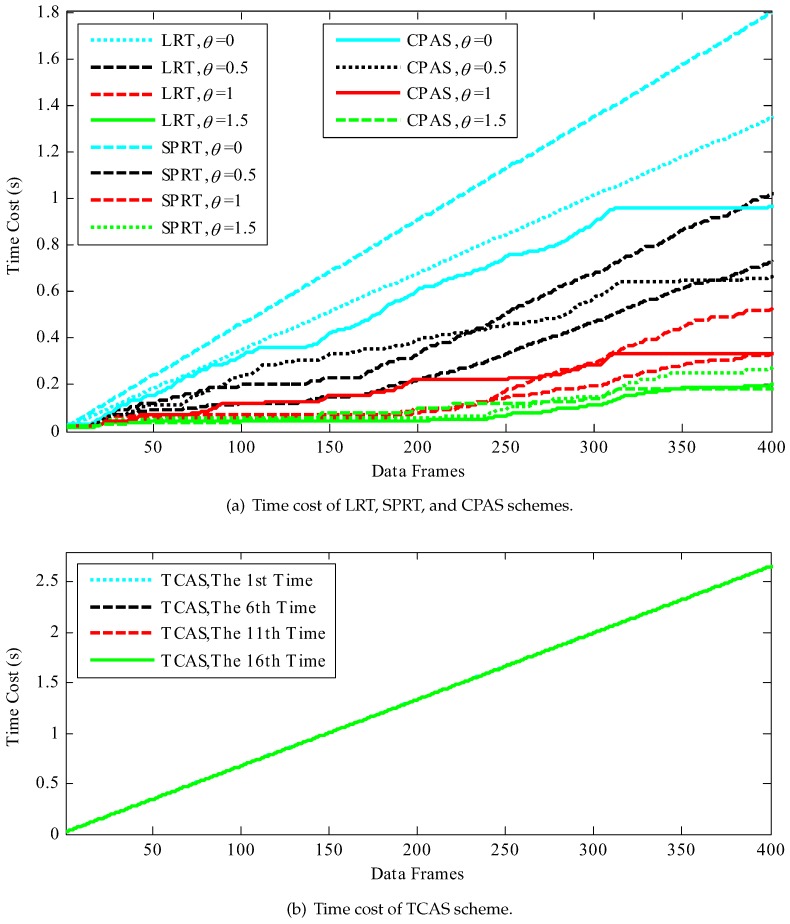
Time cost of data frames authentication, where the time cost included the physical layer channel authentication time cost (if any), upper layer cipher authentication time cost, and data demodulation time cost: (**a**) the time cost of LRT, SPRT, and CPAS schemes under different threshold values; and (**b**) the time cost of the TCAS scheme.

We considered the following performance metrics to evaluate the proposed scheme: success rate of physical-layer channel authentication, data frame loss rate, total success rate of authentication, and time cost. Success rate of physical-layer channel authentication indicates the probability of success in physical-layer channel authentication. Data frame loss rate means the ratio of the data frames lost to the data frames received by the receiver. Total success rate of authentication contains the success rate of physical-layer channel authentication and lightweight cryptographic authentication. Time cost represents the time required to authenticate data frames in simulation work, which consists of the time overhead of RC4 key initialization, physical-layer channel authentication (physical-layer channel modeling and model update also included in CPAS scheme), data demodulation, and upper layer cipher authentication. The comparative results are shown in Figure 12, Figure 13, Figure 14, Figure 15 and Figure 16. The values in the figures are all statistics in 1000 trials.

Figure 12 plots the success rate of physical-layer channel authentication at a given j=3 for varying threshold values or adjusting parameter θ. The success rates of physical-layer channel authentication gradually increased with the increasing adjusting parameter θ. When the adjusting parameter θ was high, greater than 1, the LRT, SPRT, and CPAS schemes contributed to high success rates of physical-layer channel authentication. When θ was less than 1, the success rate of physical-layer channel authentication decreased with the decreasing adjusting parameter θ. This decrease was, however, more significant in the case of the LRT and SPRT schemes. Especially, the LRT and SPRT schemes had near zero success rate of physical-layer channel authentication for θ close to zero due to each data frame received by the edge device being different, but the proposed CPAS scheme had a higher success rate due to three data frames being used to establish a physical-layer channel authentication model. Thus, the proposed scheme had a higher success rate of physical-layer channel authentication when θ was small.

Figure 13 demonstrates the comparisons among these schemes in terms of data frame loss rate. The data frame loss rate of LRT and SPRT gradually decreased with the increase of the adjusting parameter θ, while the data frame loss rate of the proposed scheme was always close to 0. It is worth noting that LRT scheme had 50% data frame loss rate and SPRT scheme had 33.3% data frame loss rate but the proposed scheme had near zero data frame loss rate when θ=0. The reason was that Bob dropped the data frame directly when the physical-layer channel authentication failed and upper layer authentication was required before each physical-layer channel authentication in the LRT and SPRT schemes. Our scheme did not discard data frames directly but activated upper layer authentication to check the validity of the data frames. Thus, no matter the value of parameter θ, the data frame loss rate of our proposed scheme was close to zero, as long as the data frame was legitimate.

Figure 14 shows the comparisons among the LRT, SPRT, and CPAS schemes in terms of total success rates of authentication, assumed to be free of attack. The total success rates of physical-layer channel authentication gradually increased with the increase of the threshold value in the LRT and SPRT schemes, while it was always close to 100% with the increase of adjusting parameter θ in the proposed scheme. The reason was that the edge device did not drop data frames directly, when physical-layer channel authentication failed, but activated upper layer authentication to verify the legality of the data frames in the CPAS scheme. This resisted losing legitimate data frames when physical-layer channel authentication failed. However, this led to some processing delay.

Figure 15 and Figure 16 plot the time costs of data frames authentication in different authentication schemes. The time costs of the LRT, SPRT, and CPAS schemes increased with the increase of the number of data frames on the whole, but decreased with the increase of threshold value. In many experiments, the time cost of traditional cipher authentication scheme (TCAS) also increased linearly with the increase of the number of data frames.

However, as evident from the results, the SPRT scheme needed more time costs than LRT and CPAS schemes when θ=0, especially with the increase of data frames. The reason was that the data frames must be demodulated before upper layer authentication. That is to say, data demodulation took more time cost before upper layer authentication, which was also a pivotal reason. In the LRT and SPRT schemes, Bob dropped the data packet directly when the physical-layer channel authentication failed and upper layer authentication was required before each physical-layer channel authentication. In the TCAS scheme, upper layer cipher authentication, which was after demodulation, was needed to verify the validity of each data frame. In the CPAS scheme, Bob did not discard data frames directly, when physical-layer channel authentication failed, but activated upper layer cipher authentication. The low time cost indicates that the CPAS scheme activated the upper layer authentication less frequently, because it had a higher successful rate of physical-layer channel authentication, when θ=0. The proposed scheme employed *j* (j=3, in our experiments) data frames to establish a physical-layer channel authentication model, which was more meaningful for practical application, and upper layer authentication to verify the legality of the data frames when physical-layer channel authentication failed.

In addition, the CPAS scheme needed more time cost than LRT and SPRT schemes with the increase of parameter θ. The low time cost also manifested that the LRT and SPRT schemes had a higher successful rate of physical-layer channel authentication when the adjusting parameter θ was large. It is worth noting that the time cost differences among the LRT, SPRT, and CPAS schemes decreased with the increase of parameter θ. Therefore, it is feasible to satisfy the requirement of the edge computing system with asymmetric resources, as long as the adjusting parameter θ is appropriate.

## 6. Conclusions

In this paper, we propose a novel cross-layer secure physical-layer authentication program for edge computing system with asymmetric resources. The proposed scheme combines clustering technology and lightweight symmetric cipher with physical-layer channel state information to achieve mutual authentication between terminals and edge devices. Theoretical analysis and experimental results show that our proposed scheme can effectively boost the total success rate of access authentication and decrease the data frame loss rate but it increases time cost slightly. It is not only secure but also simple and flexible, especially independent of a trusted party. In addition, our scheme could resist spoofing attacks, replay attacks, small integer attacks, exhaustive attacks, and guessing attacks. It can significantly reduce the access authentication complexity and achieve greater security for the edge computing system with asymmetric resources. Therefore, the proposed scheme is very suitable for the resource asymmetric authentication scenario.

## Abbreviations

The following abbreviations are used in this manuscript:

CPAS	Clustering based physical-layer authentication scheme
CPU	Central processing unit
CSI	Channel state information
ED	Edge computing device
GHz	Giga Hertz
IoT	Internet of things
LRT	Likelihood ratio test
MHz	Mega Hertz
SPRT	Sequential probability ratio test
TCAS	Traditional cipher authentication scheme
USRP	Universal software radio peripheral
